# Trends in Q fever serologic testing by immunofluorescence from four large reference laboratories in the United States, 2012–2016

**DOI:** 10.1038/s41598-018-34702-2

**Published:** 2018-11-12

**Authors:** Halie K. Miller, Alison M. Binder, Amy Peterson, Elitza S. Theel, Joseph M. Volpe, Marc Roger Couturier, Cara C. Cherry, Gilbert J. Kersh

**Affiliations:** 1 Rickettsial Zoonoses Branch, Centers for Disease Control and Prevention, Atlanta, Georgia USA; 2 Division of Clinical Microbiology, Department of Laboratory Medicine and Pathology, Mayo Clinic, Rochester, Minnesota USA; 3 Laboratory Corporation of America, Burlington, North Carolina USA; 4 Department of Pathology, University of Utah School of Medicine, Salt Lake City, Utah, USA ARUP Laboratories, Institute for Clinical and Experimental Pathology, Salt Lake City, Utah USA

## Abstract

Laboratory testing for Q fever (*Coxiella burnetii*) is essential for a differential diagnosis, yet little is known about Q fever diagnostic testing practices in the United States. We retrospectively analyzed Q fever immunoglobulin G (IgG) indirect immunofluorescence assay (IFA) testing data between 1/1/2012–10/31/2016 from ARUP, LabCorp, Mayo Medical Laboratories, and Quest Diagnostics. Data included IgG phase I and phase II titers, patient age and sex, and state and date of specimen collection. On average, 12,821 specimens were tested for Q fever annually by the participating laboratories. Of 64,106 total specimens, 84.1% tested negative for *C. burnetii*-specific antibodies. Positive titers ranged from 16 to 262,144 against both phase I and phase II antigens. Submission of specimens peaked during the summer months, and more specimens were submitted from the West North Central division. Testing occurred more frequently in males (53%) and increased with age. In conclusion, few U.S. Q fever cases are reported, despite large volumes of diagnostic specimens tested. Review of commercial laboratory data revealed a lack of paired serology samples and patterns of serology titers that differ from case reporting diagnostic criteria.

## Introduction

*Coxiella burnetii* is a gram negative, intracellular pathogen and the causative agent of Q fever. Infection typically occurs via inhalation of aerosolized particles shed from infected domestic ruminants such as goats, sheep and cattle^1,2^. Risk of exposure is increased by the ability of *C. burnetii* to persist in the air and environment^3^. *C. burnetii* has been detected in domestic animals, wildlife, marine mammals, and throughout the environment including farms, post offices, stores, and schools providing multiple sources for exposure to this organism^1,4–7^. The infectious dose for *C. burnetii* is extremely low at one to ten organisms.

In humans, Q fever is an acute febrile illness characterized by severe headache, myalgia, pneumonia, or hepatitis^1,2^. Acute Q fever is often self-limiting and as many as 60% of infections can be asymptomatic; however, in 2–5% of acute cases the disease manifests into a chronic condition often resulting in life-threatening endocarditis, vascular infection or infected aortic aneurysms^1,2^. Clinical symptoms of Q fever are indistinguishable from many other diseases, making laboratory testing essential for accurate diagnosis. The gold standard for laboratory confirmation is serological analysis to detect anti-*C. burnetii* immunoglobulin G (IgG) antibodies, typically performed using the indirect immunofluorescence assay (IFA) with two antigenically discrete phases of *C. burnetii* – phase I and phase II^8^. Phase I *C. burnetii* contains a full-length lipopolysaccharide (LPS) and antibodies against this strain typically develop to higher abundance in chronic Q fever patients. Phase II *C. burnetii*, generated from serial passages of phase I in embryonated eggs, displays a truncated LPS that lacks the O-antigen. Antibodies against phase II *C. burnetii* typically develop to higher levels during acute Q fever^9^.

Since 1999, Q fever has been a notifiable disease in the United States. As of 2008, case reports provided to the U.S. Centers for Disease Control and Prevention (CDC) have distinguished acute Q fever cases from chronic^10,11^. From 2008 to 2015, the number of annual notifications of Q fever cases to the CDC ranged from 113 to 170 cases, with annual percentages of chronic Q fever notifications ranging from 12 to 22%^12–15^. Although national surveillance provides important insight into the incidence and epidemiologic characteristics of Q fever in the United States, little is known about the diagnostic testing practices for Q fever. In an effort to better understand this, we analyzed Q fever IFA testing data collected from 1/1/2012–10/31/2016 provided by four large U.S. reference laboratories to determine the number of specimens tested, seasonal and geographical distributions, and the characteristics of serum titers from tested specimens.

## Results

### Study dataset

A total of 82,024 tests were provided by the participating laboratories from January 1, 2012 to October 31, 2016, of which 64,106 (78.2%) tests were included for analysis. The remaining 17,918 tests (21.8%) were excluded for at least one of the following reasons: (1) specimen collection date was outside of the defined study period; (2) tests had missing or indeterminate values for phase I and/or phase II titers; (3) phase I or phase II titers were not diluted to at least 1:1024; (4) results were not based on a twofold dilution series.

### Testing burden and seasonal variations in testing

**Figure 1:**
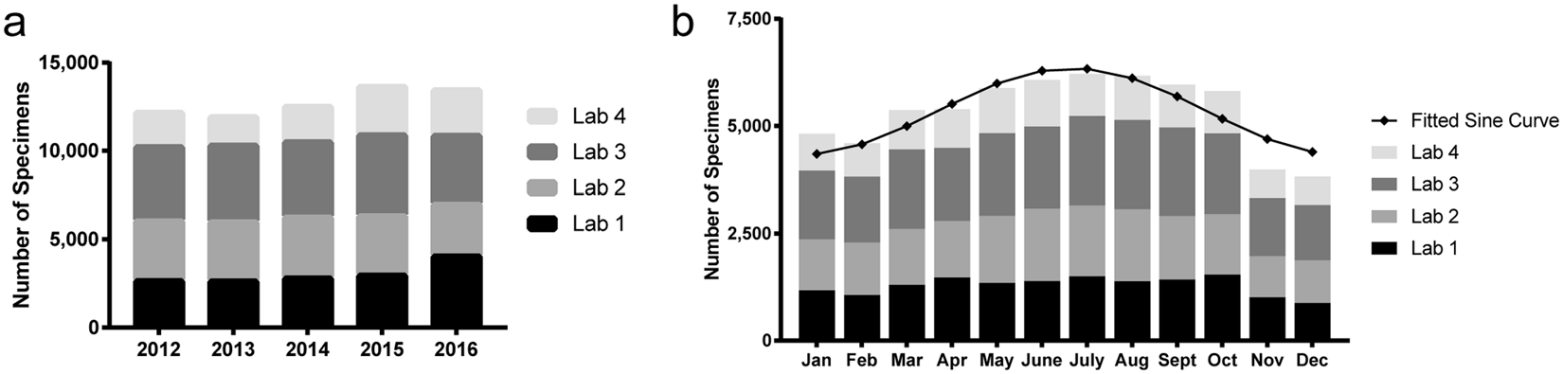
Annual volume and seasonal distribution of specimens. Total specimens tested by each laboratory are compiled based on specimen collection (**a**) year and (**b**) month.

The mean (±SD) number of specimens tested annually by the four laboratories combined was 12,821 ± 771 (Fig. 1a). The mean number of specimens tested by laboratories 1, 2, 3 and 4 were 3,099 ± 592, 3,297 ± 202, 4,242 ± 255 and 2,183 ± 476, respectively.

Total specimens tested each month across the study period followed a seasonal pattern (peak/low ratio = 1.465; 95% CI 1.432–1.499). June, July, and August had the most specimens tested with a peak in July (Fig. 1b). Similar patterns were observed for total specimens from each individual laboratory as well as for number of persons tested each month by laboratory with peaks occurring between late June and early July (data not shown).

### Characteristics of serum titers from tested specimens

Of the 64,106 specimens included for analysis, 84.1% (53,898) were negative for antibodies against both phase I and phase II *C. burnetii* (Table 1). Of the remaining 10,208 (15.9%) specimens, titers ranged from 16 to 262,144 and 5,011 (7.8%) specimens had elevated phase II titers (≥128), while 1,238 (1.9%) specimens had elevated phase 1 titers (≥1024). A total of 1,230 (1.9%) specimens had elevated titers against both phase 1 and phase 2. Of the 8 specimens that had an elevated phase 1 titer (≥1024), but a phase II titer <128, 3 were negative for antibodies against phase II. Among the 5,011 specimens with phase II titers ≥128, 3,781 (75%) had phase 1 serology below the cut-off used to support chronic Q fever diagnosis (1024), and 394 (7.9%) tested negative for antibodies against phase 1. There were 4,917 specimens (7.7% of total specimens) with phase II titers of 16, 32, or 64. These titers are below the cut-off to support acute Q fever diagnosis but may be reported as antibody detected by the reference laboratories.

**Table 1:** Serology titers of specimens.

	Titer	Phase I													Total
		<16	16	32	64	128	256	512	*1,024*	*2,048*	*4,096*	*8,192*	*16,384*	>*32,768*	
**Phase II**	<16	53,898	145	64	38	17	10	3	*1*	*1*	*1*	*0*	*0*	*0*	54,178
	16	658	547	129	98	21	7	8	*0*	*0*	*0*	*0*	*0*	*0*	1,468
	32	390	424	270	135	40	8	5	*1*	*0*	*0*	*0*	*0*	*0*	1,273
	64	472	663	330	501	124	72	10	*3*	*1*	*0*	*0*	*0*	*0*	2,176
	**128**	**183**	**295**	**243**	**344**	**258**	**124**	**28**	***9***	***3***	***0***	***0***	***0***	***0***	1,487
	**256**	**102**	**208**	**128**	**381**	**213**	**323**	**63**	***46***	***9***	***2***	***0***	***1***	***0***	1,476
	**512**	**34**	**18**	**21**	**51**	**75**	**91**	**109**	***51***	***16***	***5***	***1***	***0***	***0***	472
	**1,024**	**31**	**11**	**12**	**43**	**23**	**125**	**57**	***296***	***33***	***27***	***3***	***1***	***0***	662
	**2,048**	**23**	**4**	**4**	**9**	**16**	**20**	**24**	***51***	***97***	***45***	***12***	***2***	***0***	307
	**4,096**	**8**	**2**	**1**	**4**	**5**	**15**	**12**	***31***	***22***	***41***	***22***	***19***	***3***	185
	**8,192**	**7**	**0**	**1**	**3**	**3**	**5**	**8**	***17***	***10***	***17***	***20***	***32***	***17***	140
	**16,384**	**4**	**0**	**1**	**0**	**0**	**0**	**2**	***7***	***10***	***12***	***13***	***21***	***30***	100
	**>** **32,768**	**2**	**0**	**0**	**1**	**1**	**0**	**2**	***5***	***5***	***5***	***11***	***21***	***129***	182
**Total**		55,812	2,317	1,204	1,608	796	800	331	518	207	155	82	97	179	64,106

Titers shown in bold indicate elevated phase II serology (≥128) and those in italics indicate elevated phase I serology (≥1024). Categories are not mutually exclusive; specimens may fit both categories. A portion of the data (0.58%) are not diluted to end-point and instead are included at the highest dilution tested. All of the specimens analyzed were diluted to at least 1:1024.

Among the 5,011 specimens with phase II serology ≥128, 3,122 (62.3%) had a higher phase II titer relative to phase I; whereas, 615 (12.3%) had higher phase I titers (Table 2). For those specimens with phase I serology ≥1024, 584 (47.2%) had equal phase I and phase II titers and 408 (33.0%) had higher phase I titers, with only 246 (19.9%) displaying phase II titers greater than phase I. The geometric mean titer for specimens with phase II serology ≥128 was 230.7 (range: 16–262,144) for phase I and 491.1 (range: 128–262,144) for phase II. The geometric mean titer for specimens with phase I serology ≥1024 was 3,468.3 (range: 1024–262,144) for phase I and 3,104.2 (range: 32–262,144) for phase II. The percentage of persons receiving a second serology test within the recommended time frame ranged from 0.69% to 3.0% for the four laboratories.

**Table 2:** Laboratory characteristics of specimens.

	Ph2 > 128 (n = 5,011)		Ph1 > 1024 (n = 1,238)	
	n	%	n	%
**Serologic characteristics**				
Phase I titer < phase II titer	3,122	62.3	246	19.9
Phase I titer = phase II titer	1,274	25.4	584	47.2
Phase I titer > phase II titer	615	12.3	408	33.0
**Geometric mean titer (range)**				
Phase I GMT (range)	230.7 (16–262,144)		3,468.3 (1024–262,144)	
Phase II GMT (range)	491.1 (128–262,144)		3,104.2 (32–262,144)	

Specimens with titers <16 were excluded from GMT calculations. A portion of the data (0.58%) are not diluted to end-point and can lead to overestimation of the PhI = PhII category and underestimation of the GMT.

### Geographical distribution of specimens

The state, territory, or district of specimen submission was reported for nearly all specimens included for analysis (63,952; 99.8%), representing all 50 states, Puerto Rico, and the District of Columbia (Table 3). The fewest submissions came from Hawaii (7; 0.01%), whereas the greatest number of submissions came from Texas (6,877; 10.8%). By U.S. census division, the New England division (Connecticut, Maine, Massachusetts, New Hampshire, Rhode Island, and Vermont) had the fewest submissions (2,726; 4.3%) and the West North Central division (WNCD; Iowa, Kansas, Minnesota, Missouri, Nebraska, North Dakota, and South Dakota) had the largest number of specimens tested (10,455; 16.3%). Adjusting for population, Alaska had the fewest submissions in this data set, with 4.5 per million persons (PMP) and South Dakota had the most (1,820 PMP) (Table 3).

**Table 3:** Q fever serology by division and state.

U.S. Census Division and State	Total Tests (n = 63,952)			Ph2 > 128 (n = 4,998)			Ph1 > 1024 (n = 1,233)			Total elevated serology (n = 5,006)		
	n	per capita^a^	%	n	per capita^a^	%^b^	n	per capita^a^	%^b^	n	per capita^a^	%^b^
**New England**	**2726**	**185.8**	**4.3**	**176**	**12.0**	**6.5**	**13**	**0.9**	**0.5**	**176**	**12.0**	**6.5**
Connecticut	420	117.0	0.7	42	11.7	10.0	2	0.6	0.5	42	11.7	10.0
Maine	247	185.7	0.4	5	3.8	2.0	1	0.8	0.4	5	3.8	2.0
Massachusetts	1583	234.8	2.5	79	11.7	5.0	5	0.7	0.3	79	11.7	5.0
New Hampshire	126	94.9	0.2	5	3.8	4.0	1	0.8	0.8	5	3.8	4.0
Rhode Island	119	112.9	0.2	11	10.4	9.2	0	0.0	0.0	11	10.4	9.2
Vermont	231	368.9	0.4	34	54.3	14.7	4	6.4	1.7	34	54.3	14.7
**Middle Atlantic**	**9702**	**234.4**	**15.2**	**360**	**8.7**	**3.7**	**56**	**1.4**	**0.6**	**360**	**8.7**	**3.7**
New Jersey	5657	634.5	8.8	107	12.0	1.9	20	2.2	0.4	107	12.0	1.9
New York	2759	140.1	4.3	165	8.4	6.0	32	1.6	1.2	165	8.4	6.0
Pennsylvania	1286	100.6	2.0	88	6.9	6.8	4	0.3	0.3	88	6.9	6.8
**E. N. Central**	**8060**	**172.6**	**12.6**	**557**	**11.9**	**6.9**	**107**	**2.3**	**1.3**	**559**	**12.0**	**6.9**
Illinois	1699	132.2	2.7	80	6.2	4.7	15	1.2	0.9	81	6.3	4.8
Indiana	592	89.8	0.9	18	2.7	3.0	4	0.6	0.7	18	2.7	3.0
Michigan	2382	240.4	3.7	117	11.8	4.9	13	1.3	0.5	117	11.8	4.9
Ohio	1882	162.4	2.9	131	11.3	7.0	37	3.2	2.0	131	11.3	7.0
Wisconsin	1505	261.5	2.4	211	36.7	14.0	38	6.6	2.5	212	36.8	14.1
**W. N. Central**	**10455**	**498.2**	**16.3**	**999**	**47.6**	**9.6**	**330**	**15.7**	**3.2**	**1001**	**47.7**	**9.6**
Iowa	1472	473.8	2.3	147	47.3	10.0	83	26.7	5.6	148	47.6	10.1
Kansas	1403	484.1	2.2	87	30.0	6.2	22	7.6	1.6	87	30.0	6.2
Minnesota	2646	485.4	4.1	237	43.5	9.0	54	9.9	2.0	238	43.7	9.0
Missouri	2032	335.3	3.2	155	25.6	7.6	34	5.6	1.7	155	25.6	7.6
Nebraska	928	493.3	1.5	70	37.2	7.5	15	8.0	1.6	70	37.2	7.5
North Dakota	425	577.3	0.7	32	43.5	7.5	20	27.2	4.7	32	43.5	7.5
South Dakota	1549	1820.1	2.4	271	318.4	17.5	102	119.9	6.6	271	318.4	17.5
**S. Atlantic**	**7568**	**121.1**	**11.8**	**287**	**4.6**	**3.8**	**66**	**1.1**	**0.9**	**287**	**4.6**	**3.8**
Delaware	94	100.6	0.1	3	3.2	3.2	0	0.0	0.0	3	3.2	3.2
District of Columbia	205	311.1	0.3	11	16.7	5.4	0	0.0	0.0	11	16.7	5.4
Florida	2777	139.3	4.3	94	4.7	3.4	6	0.3	0.2	94	4.7	3.4
Georgia	781	77.3	1.2	47	4.7	6.0	24	2.4	3.1	47	4.7	6.0
Maryland	614	103.0	1.0	26	4.4	4.2	2	0.3	0.3	26	4.4	4.2
North Carolina	1167	117.4	1.8	55	5.5	4.7	16	1.6	1.4	55	5.5	4.7
South Carolina	332	68.7	0.5	8	1.7	2.4	0	0.0	0.0	8	1.7	2.4
Virginia	1394	167.7	2.2	34	4.1	2.4	18	2.2	1.3	34	4.1	2.4
West Virginia	204	110.5	0.3	9	4.9	4.4	0	0.0	0.0	9	4.9	4.4
**E. S. Central**	**2973**	**158.2**	**4.6**	**137**	**7.3**	**4.6**	**47**	**2.5**	**1.6**	**137**	**7.3**	**4.6**
Alabama	536	110.7	0.8	11	2.3	2.1	1	0.2	0.2	11	2.3	2.1
Kentucky	745	168.9	1.2	51	11.6	6.8	22	5.0	3.0	51	11.6	6.8
Mississippi	329	110.1	0.5	10	3.3	3.0	8	2.7	2.4	10	3.3	3.0
Tennessee	1363	208.2	2.1	65	9.9	4.8	16	2.4	1.2	65	9.9	4.8
**W. S. Central**	**8389**	**231.6**	**13.1**	**573**	**15.8**	**6.8**	**151**	**4.2**	**1.8**	**573**	**15.8**	**6.8**
Arkansas	732	993.4	1.1	50	67.9	6.8	6	8.1	0.8	50	67.9	6.8
Louisiana	385	82.9	0.6	6	1.3	1.6	1	0.2	0.3	6	1.3	1.6
Oklahoma	395	101.9	0.6	37	9.5	9.4	17	4.4	4.3	37	9.5	9.4
Texas	6877	255.1	10.8	480	17.8	7.0	127	4.7	1.8	480	17.8	7.0
**Mountain**	**5611**	**288.6**	**8.8**	**631**	**32.5**	**11.2**	**167**	**8.6**	**3.0**	**634**	**32.6**	**11.3**
Arizona	1408	474.3	2.2	82	27.6	5.8	21	7.1	1.5	82	27.6	5.8
Colorado	2068	385.9	3.2	183	34.1	8.8	67	12.5	3.2	185	34.5	8.9
Idaho	66	40.4	0.1	13	7.9	19.7	0	0.0	0.0	13	7.9	19.7
Montana	314	306.8	0.5	32	31.3	10.2	17	16.6	5.4	32	31.3	10.2
Nevada	188	66.2	0.3	18	6.3	9.6	3	1.1	1.6	18	6.3	9.6
New Mexico	381	182.9	0.6	41	19.7	10.8	17	8.2	4.5	42	20.2	11.0
Utah	1093	370.7	1.7	230	78.0	21.0	18	6.1	1.6	230	78.0	21.0
Wyoming	93	159.5	0.1	32	54.9	34.4	24	41.2	25.8	32	54.9	34.4
**Pacific**	**8364**	**144.6**	**13.1**	**1270**	**22.0**	**15.2**	**293**	**5.1**	**3.5**	**1271**	**22.0**	**15.2**
Alaska	30	4.5	0.05	4	0.6	13.3	0	0.0	0.0	4	0.6	13.3
California	5739	148.5	9.0	1100	28.5	19.2	248	6.4	4.3	1100	28.5	19.2
Hawaii	7	5.0	0.01	3	2.1	42.9	0	0.0	0.0	3	2.1	42.9
Oregon	764	191.9	1.2	73	18.3	9.6	20	5.0	2.6	73	18.3	9.6
Washington	1824	257.9	2.9	90	12.7	4.9	25	3.5	1.4	91	12.9	5.0
**Territories**	**24**	**6.8**	**0.04**	**4**	**1.1**	**16.7**	**0**	**0.0**	**0.0**	**4**	**1.1**	**16.7**
Puerto Rico	24	6.8	0.04	4	1.1	16.7	0	0.0	0.0	4	1.1	16.7

Only specimens with geographical information were included. Specimens likely represent the location of the submitting physician and are not necessarily reflective of the patient’s state of residence.

^a^Per 1,000,000 persons, based on US Census data.

^b^Percentage relative to total tests performed in each respective division or state.

Elevated phase 2 titers (≥128) were most commonly observed in the Pacific census division (Alaska, California, Hawaii, Oregon, and Washington). This division had 1270 specimens with phase 2 titers ≥128, mostly due to the 1100 specimens from California. The WNCD had the most specimens (330) with elevated phase 1 titers (≥1024), and California was the state with the most specimens with phase 1 ≥1024. Adjusting for population, South Dakota had the highest rate of phase 2 titers ≥128 (318.4 PMP), and phase 1 titers ≥1024 (119.9 PMP) in this dataset.

### Characteristics of persons tested for Q fever by lab

Demographic characteristics of persons tested were summarized for each laboratory. During the study period, laboratory 1, 2, 3 and 4 averaged testing of 2,744 ± 536 (min/max 2,367/3,683), 3,064 ± 186 (min/max 2,743/3,090), 3,750 ± 237 (min/max 3,358/3,977) and 1,594 ± 216 (min/max 1,278/1,513) persons per year, respectively (Table 4). The majority of persons had only a single test performed by a given laboratory, with 12,686 (92.5%) persons tested once by laboratory 1, and 14,578 (95.2%), 17,475 (93.2%), and 6,540 (82.1%) persons tested once by laboratories 2, 3, and 4. Of those tested at least twice, the mean number of tests per person ranged from 2.6 at laboratory 2 to 3.1 at laboratory 4. The number of tests per person went as high as 45. The median number of days between first and last tests ranged from 103 (min/max 1/1,730) at laboratories 1 and 3 to 206.5 (min/max 1/1,745) at laboratory 4.

**Table 4:** Persons tested for Q fever by lab.

	Lab 1 (n = 13,722)		Lab 2 (n = 15,320)		Lab 3 (n = 18,751)		Lab 4 (n = 7,970)	
	n	%	n	%	n	%	n	%
**#persons tested**								
2012	2,607	19.0	3,185	20.8	3,844	20.5	1,604	20.1
2013	2,367	17.3	3,090	20.2	3,849	20.5	1,278	16.0
2014	2,444	17.8	3,199	20.9	3,723	19.9	1,513	19.0
2015	2,621	19.1	3,103	20.3	3,977	21.2	1,841	23.1
2016	3,683	26.8	2,743	17.9	3,358	17.9	1,734	21.8
Average	2,744	—	3,064	—	3,750	—	1,594	—
St Dev	536	—	186	—	237	—	216	—
**Sex**								
Male	7,611	55.5	7,291	47.6	10,154	54.2	4,235	53.1
Female	6,040	44.0	8,014	52.3	8,553	45.6	3,709	46.5
Unknown	71	0.5	15	0.1	44	0.2	26	0.3

Percentages may not equal 100 due to rounding.

Males accounted for the majority of persons tested by laboratory 1 with 7,611 (55.5%) total across the study period relative to 6,040 (44.0%) females. The same was true for laboratories 3 and 4 with 10,154 (54.2%) and 4,235 (53.1%) males, respectively. Conversely, laboratory 2 tested more females during the study period with 8,014 females tested (52.3%) versus 7,291 (47.6%) males.

**Figure 2:**
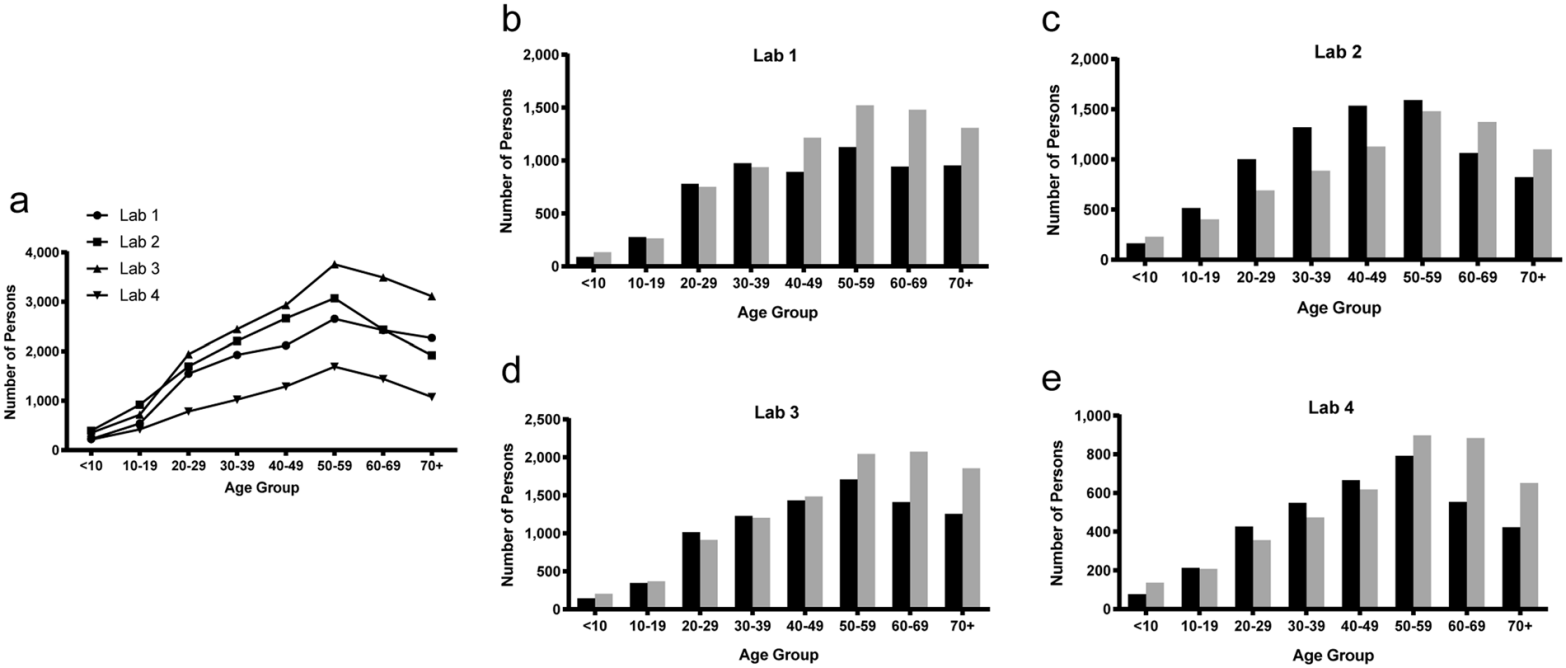
Trends in age and sex of persons tested by lab. (**a**) Total persons tested by each laboratory were analyzed based on age at the time of first sample collection. (**b–e**) Sex of persons tested by each individual laboratory (1–4) were analyzed by age group. Males are represented by grey bars, females by black bars.

The age group with the most persons tested for each of the four laboratories was 50 to 59 years (Fig. 2a). In three of the four laboratories there were more males tested at age ≥50 years relative to females (Fig. 2b–e). Laboratory 2 had more males tested than females at age ≥60 years. For laboratories 2 and 4, more females were tested between the ages of 10 to 59 years and 20 to 49 years, respectively.

## Discussion

We used reference laboratory testing data to better understand diagnostic testing practices for Q fever in the United States. On average 12,821 serology specimens were tested for Q fever by IFA annually by participating laboratories from 2012 to 2016. When considering the other laboratories offering Q fever IFA testing not included in this study (such as other reference laboratories, clinical or academic laboratories, laboratories associated with the Department of Veteran Affairs or Department of Defense) coupled with the knowledge that other Q fever diagnostic tests are available such as ELISA, PCR and immunohistochemical methods, the true volume of specimens analyzed for Q fever annually is higher. Regardless, the number of specimens tested for Q fever is surprising considering only 113–170 Q fever case reports are received by the CDC annually through passive surveillance^12–15^. Although it is likely that a portion of the specimens are from repeat testing of persons, as our study could not capture overlap among the laboratories, our study identified between 1,594 and 3,750 persons tested each year by the individual laboratories, of which, 82.5% to 95.2% were only tested by the laboratory once. The majority of specimens in this study tested negative for anti-*C. burnetii* antibodies. While the majority of specimens tested were negative, paired acute and convalescent specimens are required to accurately diagnose Q fever. The number of tests for Q fever here could indicate that physicians are considering Q fever as a differential diagnosis. When Q fever is considered, paired specimens should be submitted.

Antibodies against *C. burnetii* typically develop 7–15 days after symptoms appear; therefore, a person early in acute disease can present with an initial negative serology followed by the appearance of antibodies in the convalescent sample taken 3–6 weeks later^8^. The percentage of persons receiving a second serology test within the recommended time frame ranged from 0.69% to 3.0% for the four laboratories. Conversely, persons tested only once by each laboratory ranged from 82.1% to 95.2%. While it is possible that persons received additional testing from other laboratories or outside of the study time period, there remains a large proportion of patients represented by the reported specimens who only received a single test. In the absence of follow-up specimens to demonstrate the change in titer over time, a number of cases may go undiagnosed. Testing of a convalescent specimen should be performed even when an initial titer is negative. Further investigation into testing practices will potentially lead to improvement of these practices as well as better diagnosis and reporting of cases.

Passive surveillance data indicates incidence of acute Q fever is highest during late spring and early summer, following the birthing season of many ruminants and corresponding with an increase in outdoor activities^16^. Similarly, we observed a peak in testing of specimens for Q fever by IFA during the summer, the same pattern observed for the number of persons tested each month by laboratory. In this study, three of the four participating laboratories tested more males than females and all four laboratories tested more males over the age of 60 years. Furthermore, all participating laboratories demonstrated increased testing of persons as age increased. Passive surveillance data shows that the incidence of Q fever is higher in males and increases with age with a peak at 60–64 years old^16^. It is unclear whether seasonality and demographics of persons tested for Q fever influences the number of confirmed cases captured by passive surveillance. Alternatively, the current clinical understanding of at risk groups for Q fever disease may influence current testing practices; however, in order to address this, further studies are needed.

The current paradigm for Q fever serology is that serum from acute Q fever patients has higher phase II titers relative to phase I, while serum from chronic patients has higher phase I titers relative to phase II. Indeed, the CSTE 2009 case definition for Q fever requires the phase I titer to be higher than phase II for diagnosis of chronic Q fever^10,11^. Our analysis of serology titers from the reference laboratories suggests that serology may often deviate from these paradigms. Very few specimens (0.04%) had phase I titers ≥1024 and low phase II titers (<128). Conversely, phase II titers were shown to reach values of 32,768 or more with no detectible phase I titer. Further, a large portion of samples from this study, 45.9%, with phase I titers ≥1024 have equivalent phase II titers. Despite elevated titers against phase I *C. burnetii*, serology results from these specimens would not be used as laboratory confirmation of chronic Q fever.

The findings in this study are limited by the inability to capture data from all laboratories offering testing for Q fever in the United States. Additionally, although IFA is the gold standard for Q fever diagnosis, other laboratory tests are available including PCR, immunohistochemical methods, *C. burnetii* isolation and additional serology based assays; therefore, we are unable to capture all the Q fever testing that occurs in the United States. Due to the subjective nature of the fluorescent readouts, IFA results can vary between technicians; therefore, antibody titers may not be consistent across laboratories. Furthermore, we cannot rule out the possibility of inaccuracies. Due to the use of de-identified data in this study, we are unable to identify patients who may have received testing from multiple laboratories. Furthermore, we cannot eliminate the possibility that specimens from the same patient were sent to different laboratories within certain regions where testing may occur more frequently by one or more laboratories. Region of test may not represent region where people reside. Therefore, we may over or under ascribe region based on the data we received.

In conclusion, the findings suggest a large number of specimens are tested for Q fever by IFA annually and the overwhelming majority are negative for *C. burnetii* antibodies. Very few specimens submitted had a second sample associated with the first sample, suggesting that a portion of these negative specimens may be from undiagnosed cases. The pattern of serology titers amongst the specimens suggests that the strict paradigm of phase 2 > phase 1 = acute disease and phase 1 > phase 2 = chronic disease is an oversimplification. The data also demonstrate that more specimens are submitted for testing during the summer months, which follows the seasonal pattern for incidence of Q fever. States within the West North Central division submitted the most specimens for Q fever testing, and states from the Pacific division had the most specimens with elevated titers. Finally, our data demonstrates that demographic characteristics of persons tested for Q fever aligned with demographics of persons from reported cases through national passive surveillance.

## Methods

We performed a retrospective cross-sectional study analyzing longitudinal, de-identified data for Q fever IgG IFA tests ordered by healthcare providers in the U.S. from January 1, 2012 to October 31, 2016 at four large reference laboratories. Collaborating laboratories included ARUP, LabCorp and Mayo Medical Laboratories. Data was purchased from Quest Diagnostics. Data provided from each lab included unique identifiers to distinguish between specimens originating from the same person, IgG serology titers against phase I and phase II *C. burnetii*, age at the time of each specimen collection, sex, and state and date of specimen collection. It was not possible to determine whether persons were tested by more than one laboratory prior to or during the study period.

### Approval

This study was deemed research not involving human subjects under 45 CFR 46.102(f) according to the CDC Human Research Protection Office.

### Data analyses

Unique identifiers were modified to differentiate specimens tested by each laboratory. Data was compiled across all participating laboratories and specimens were excluded from further analysis unless both phase I and phase II titer values were known and diluted to at least 1:1024 as determined by a standard two-fold serial dilution series.

Seasonal variation in total specimens tested by collection month was evaluated based on Edwards method, a geometrical model fitting the monthly counts to a sine curve, using Episheet^17^. Serum titers were characterized based on the pattern of the phase I titer relative to phase II. An elevated phase II titer was defined as ≥128 based on the Council of State and Territorial Epidemiologist (CSTE) 2009 case definition for laboratory supportive evidence of acute Q fever from a single IFA IgG titer^10,11^. An elevated phase I titer was defined as ≥1024 based on both the CSTE 2009 case definition for laboratory confirmed criteria of chronic Q fever (≥800 IgG by IFA) and the Dutch consensus guidelines for possible chronic Q fever (≥1024 IgG by IFA)^10,11,18^. To calculate the geometric mean titer (GMT), titers were transformed by taking the log (base 2) of the titer. GMT was then determined by raising 2 to the power of the arithmetic mean of the transformed data. GMT calculations include only titers ≥16 and in cases where the titer was not determined to endpoint, the highest dilution available was used.

Person-level data were analyzed separately for each participating laboratory. The number of persons tested based on collection year of the first specimen submitted was determined. Data was analyzed using SAS version 9.4 (SAS Institute Inc., Cary, North Carolina) and GraphPad Prism version 7.01 (GraphPad Software Inc., La Jolla, California).

### Disclaimer

The findings and conclusions in this report are those of the authors and do not necessarily represent the views of the CDC.

## Data Availability

The data that support the findings of this study are available from the respective laboratories, ARUP, LabCorp, Mayo Medical Laboratories, and Quest Diagnostics, but restrictions apply to the availability of these data, which were used under agreements for the current study, and so are not publicly available. Data are however available from the authors upon reasonable request and with the permission of ARUP, LabCorp, Mayo Medical Laboratories and Quest Diagnostics.
